# A member of the *TERMINAL FLOWER 1/CENTRORADIALIS* gene family controls sprout growth in potato tubers

**DOI:** 10.1093/jxb/ery387

**Published:** 2018-11-03

**Authors:** Wayne L Morris, M Carmen Alamar, Rosa M Lopez-Cobollo, Javier Castillo Cañete, Mark Bennett, Jeroen Van der Kaay, Jennifer Stevens, Sanjeev Kumar Sharma, Karen McLean, Andrew J Thompson, Leon A Terry, Colin G N Turnbull, Glenn J Bryan, Mark A Taylor

**Affiliations:** 1 The James Hutton Institute, Invergowrie, Dundee, UK; 2 Plant Science Laboratory, Cranfield University, Bedfordshire, UK; 3 Department of Life Sciences, Imperial College London, London, UK

**Keywords:** Abscisic acid, cytokinin, dormancy, potato, sprouting, storage, *TERMINAL FLOWER 1/ CENTRORADIALIS*

## Abstract

Potato tuber bud dormancy break followed by premature sprouting is a major commercial problem which results in quality losses and decreased tuber marketability. An approach to controlling premature tuber sprouting is to develop potato cultivars with a longer dormancy period and/or reduced rate of sprout growth. Our recent studies using a potato diploid population have identified several quantitative trait loci (QTLs) that are associated with tuber sprout growth. In the current study, we aim to characterize a candidate gene associated with one of the largest effect QTLs for rapid tuber sprout growth on potato chromosome 3. Underlying this QTL is a gene encoding a TERMINAL FLOWER 1/CENTRORADIALIS homologue (PGSC0003DMG400014322). Here, we use a transgenic approach to manipulate the expression level of the *CEN* family member in a potato tetraploid genotype (cv. Désirée). We demonstrate a clear effect of manipulation of *StCEN* expression, with decreased expression levels associated with an increased rate of sprout growth, and overexpressing lines showing a lower rate of sprout growth than controls. Associated with different levels of *StCEN* expression were different levels of abscisic acid and cytokinins, implying a role in controlling the levels of plant growth regulators in the apical meristem.

## Teaser

Transgenic experiments demonstrate that TERMINAL FLOWER 1/CENTRORADIALIS is associated with potato tuber sprout growth during storage, and with the regulation of cytokinin and ABA levels.

## Introduction

Potato is the third most important food crop in the world after rice and wheat. More than a billion people worldwide eat potatoes, and global crop production exceeds 300 Mt per annum (Birch *et al.*, 2012). For both fresh and processing sectors, there is a year-round demand for potato tubers, leading to a requirement for long-term post-harvest storage on an industrial scale. The maintenance of post-harvest quality is of vital importance to producers and processors. Potato tuber bud dormancy break followed by premature sprouting is a major commercial problem which results in quality losses and reduced tuber marketability.

The length of the dormancy period is under genetic, physiological, hormonal, and environmental control (reviewed in Sonnewald and Sonnewald, 2014). In summary, abscisic acid (ABA) has a role in establishing dormancy, cytokinins are required for endodormancy break, and gibberellins and ethylene have opposing roles in sprout extension. The role of apocarotenoids other than ABA, such as strigolactones, is emerging. For example, down-regulation of the potato *CCD8* gene reduces strigolactone content, resulting in an increased rate of tuber sprouting (Pasare *et al.*, 2013). CIPC [isopropyl-*N*-(3-chlorophenyl) carbamate] is the most frequently used sprout suppressant for potatoes, and can be effective for long-term (up to 9 months) storage at temperatures between 8 °C and 12 °C (Campbell *et al.*, 2010; Paul *et al.*, 2016). However, CIPC has been under severe controls in recent years with the establishment of a maximum residue limit of 10 mg kg^−1^ (European Annex I clearance under Directive EC/91/414). Exogenous ethylene supplementation has been presented as an alternative sprout suppressant method as, although it terminates endodormancy, it prolongs ecodormancy and inhibits sprout elongation (Foukaraki *et al*., 2016*a*, *b*). Other chemical treatments have also been identified for the control of tuber sprouting (reviewed in Alamar *et al.*, 2017), but currently there is limited adoption.

A parallel approach to controlling premature tuber sprouting is to develop potato cultivars with a longer dormancy period and/or reduced rate of sprout growth. Several studies have demonstrated considerable genetic variation within potato germplasm for traits related to tuber sprout growth. For example, van den Berg *et al.* (1996) found quantitative trait loci (QTLs) on nine chromosomes involved in the regulation of tuber dormancy, both separately and with epistatic effects. Other genetic studies have added to this knowledge including our recent studies using a potato diploid population where several QTLs that are associated with tuber sprout growth were identified. In the current study, we characterized a candidate gene associated with one of the largest effect QTLs for rapid tuber sprout growth on potato chromosome 3. Underlying this QTL is a gene encoding a TERMINAL FLOWER 1/CENTRORADIALIS orthologue (PGSC0003DMG400014322), a member of the 13 gene *FT* gene family identified in the potato genome (Abelenda *et al.*, 2014), subsequently referred to as *StCEN* herein.

Previous studies in poplar trees have identified that a *CEN* family member plays an important role in the regulation of maturity, in maintaining vegetative identity, and in the transition from bud dormancy to shoot growth. Mohamed *et al.* (2010) found that reduced expression of *PopCEN1* in transgenic poplars (*Populus* spp.) shortened dormancy release and accelerated the acquisition of traits related to maturity (first flowering, inflorescence number, and proportion of short shoots), whereas overexpressing *PopCEN1* trees showed opposite effects and, in some transgenic lines, complete inhibition of flowering. Huang *et al.* (2012) also observed the inhibition of floral initiation by an *Arabidopsis thaliana CENTRORADIALIS* homologue (ATC). Varkonyi-Gasic *et al*. (2013) suggest roles for *FT*, *CEN*, and *FD* in integration of developmental and environmental cues that affect dormancy, budbreak, and flowering in kiwifruit. Here we use a transgenic approach to manipulate the expression level of a potato *CEN* gene associated with a tuber sprouting QTL in a potato tetraploid genotype (cv. Désirée) and demonstrate clear effects on tuber bud hormone content and sprout growth.

## Materials and methods

### Generation of StCEN transgenic potato lines

A homologue of *TERMINAL FLOWER 1/CENTRORADIALIS* (gene identifier PGSC0003DMG400014322, transcript number PGSC0003DMT400037143) was identified as a candidate gene underlying a large effect QTL for tuber sprouting on potato chromosome 3 (S.K. Sharma *et al*., unpublished). To generate *StCEN* constructs for overexpressing transgenic potato lines, primers were designed to amplify a 528 bp ORF from tuber cDNA prepared from *Solanum tuberosum* cv. Désirée. *Sal*I sites were engineered at both termini to facilitate subcloning into *Sal*I-digested pB19_35S, and restriction mapping was used to identify constructs with *StCEN* in the sense orientation. pB19_35S was created in-house by transferring the *Kpn*I/*Xho*I-digested expression cassette of pJIT60 [identical to pJIT30 (Guerineau *et al.*, 1990), but with a double rather than a single 35S *Cauliflower mosaic virus* (CaMV) promoter] into *Kpn*I/*Sal*I-digested pBIN19 (Bevan, 1984). *StCEN*-down-regulated transgenic potato lines were generated utilizing the Golden Gate-based RNAi vector pRNAi-GG (Yan *et al.*, 2012). A 442 bp *StCEN*-specific fragment was amplified by PCR and used in a restriction–ligation reaction for insertion into the pRNAi-GG vector. Details of primer sequences used for cloning are provided in Supplementary Table S1 at *JXB* online. The resulting binary vectors were transformed into *Agrobacterium tumefaciens* strain AGL1 by electroporation. Transformed cells were selected for their resistance to kanamycin and rifampicin. *Agrobacterium*-mediated potato transformation (cv. Désirée) was performed as described previously (Ducreux *et al.*, 2005).

### Plant material and growth conditions

Tissue culture plantlets were propagated in 90 mm Petri dishes containing Murashige and Skoog (MS) medium (Murashige and Skoog, 1962) supplemented with 20 g l^−1^ sucrose and 8 g l^−1^ agar at 18 ± 4 °C, 16 h light, 8 h dark, light intensity 100 µmol m^−2^ s^−1^. Four weeks after subculture *in vitro*, plantlets were transferred to 12 cm pots containing compost and grown in a glasshouse, under conditions of 16 h light (18 °C) and 8 h dark (15 °C). Light intensity ranged from 400 µmol m^−2^ s^−1^ to 1000 µmol m^−2^ s^−1^ at canopy height. After 20 weeks, tubers were harvested and stored at 4 °C or assessed for tuber sprout growth.

### Assessment of tuber sprout growth

Tubers from multiple independent transgenic lines were assessed for tuber sprout growth. Following harvest, tubers were placed in darkness for 2 weeks at 4 °C, then were transferred to a controlled-environment growth chamber set at 20 °C and 65% relative humidity (RH). The longest sprouts from six individual tubers from each transgenic line were measured weekly for a period of 10 weeks.

### RNA extraction and quantitative real-time PCR (RT-PCR)

RNA was extracted from potato tuber buds excised from tubers after 5 months of storage at 4 °C as described (Ducreux *et al.*, 2008). The first-strand cDNA templates were generated by reverse transcription, using random hexamers as primer and SuperScript II reverse transcriptase (Invitrogen Life Technologies, Carlsbad, CA, USA). Potato elongation factor1-alpha (*EF1α*) primers were used as a control. The expression level of St*CEN* was determined using the StepOnePlus Real-Time PCR system (Applied Biosystems) and StepOne Software version 2.3 (Applied Biosystems). Gene-specific primers and Universal probe Library (UPL, Roche Life Science) probes (Supplementary Table S1) were used at a concentration of 0.2 µM and 0.1 µM, respectively. Thermal cycling conditions were: 10 min denaturation at 95 °C followed by 40 cycles of 15 s at 94 °C, and 60 s at 60 °C. Relative expression levels were calculated and the primers validated using the Ct method (Livak and Schmittgen, 2001). To normalize the values, an alternative method for calculating relative quantification was used (Pfaffl, 2001). For greater sensitivity, buds from *StCEN* RNAi lines and controls were analysed using a QuantiTect SYBR^®^ green PCR kit (Qiagen) according to the manufacturer’s instructions.

### Detailed characterization of tuber sprouting in selected transgenic lines

Based on preliminary assessment of tuber sprout growth and the *StCEN* expression level, four transgenic lines (two overexpressing lines and two RNAi lines with reduced expression) and a wild-type control were grown from a subsequent vegetative generation. The phenotype was confirmed and the time of dormancy break and changes in plant growth regulator levels in buds during storage were characterized in detail.

A randomized experiment was designed for the five genotypes—for each genotype, five tubers from three individual plants were studied at each sampling point (week 0, 1, 3, 5, and 7 post-harvest) for detailed analysis of dormancy break. At each sampling point, ethylene production and tuber respiration rate were measured, followed by a dormancy break assessment to observe bud development, and finally sample preparation, in which the apical buds were excised from the tubers for further biochemical analysis. Tubers from each line were placed in a tray for easy manipulation and held at 20 ± 1 °C under an RH that ranged between 55% and 60%. Temperature and RH were recorded constantly using wireless sensors (RDSens, Prodisei Technologies, Spain)

### Physiological assessment: endogenous ethylene production and respiration rate

Ethylene production was analysed at each sampling point using a laser-based ethylene detector (EDT-300 real time sub-ppb ethylene analyser, Sensor Sense BV, Nijmegen, The Netherlands) consisting of a CO_2_ laser and a photoacoustic cell. For each sampling day, tuber samples were placed in glass cuvettes (with an empty jar as control) and at a constant air flow of 4 l h^−1^. Samples were incubated for 30 min in the cuvettes before the ethylene (nl h^−1^) produced by the tubers was measured by automatic integration with EDT-software using ‘stop and flow’ mode. Final ethylene measures were expressed as nl h^−1^ kg^−1^.

Respiration rate, expressed as CO_2_ production (ml h^−1^ kg^−1^), was analysed using a Sable Respirometer System (Model 1.3.8 Pro, Sable Systems International, NV, USA) with O_2_ calibrated in the range 19–21.5% and CO_2_ in the range 0–1% (Collings *et al*., 2012). The tubers were placed into hermetic jars connected to a compressed air bottle that provided a constant flow of 300 ml min^−1^, regulated by a flow measurement system so as to avoid the development of a modified atmosphere. All measurements were performed in triplicate (five tubers per replicate).

### Dormancy phenotyping

Tuber apical buds were assessed each week using a binocular stereomicroscope. The times to dormancy break and sprout growth were assessed according to five different stages that were defined from deep dormancy to fully sprouting (Supplementary Fig. S1). In the first stage, the meristematic tissue is completely covered by primordial leaves [dormant (D)]; in stage two the meristem starts to emerge, [pre-eye movement (PEM)]; at stage three dormancy is broken when very small white buds start being clearly visible [eye movement (EM): <1 mm); at stage four no protecting leaves are left and the white buds are more developed [small sprout (SS): 1–2 mm); finally, when the sprouts are >2 mm, tubers are deemed to be at the fifth stage [sprout (S)]. In this last stage, the sprout length was also assessed in order to quantify differences in sprout growth/vigour between genotypes.

### Hormone analysis

Apical and adjacent tuber buds were excised at each sampling point (in triplicate and from five tubers per replicate) using a scalpel, while under a stereomicroscope, and immediately snap-frozen in liquid nitrogen prior to storage at –80 °C. Samples were lyophilised in a freeze-dryer (Scanvac, Lynge, Denmark) in the dark for 3 d.

Freeze-dried samples were weighed and powdered using a TissueLyser (Model II, QIAGEN, Hilden, Germany) for 2 min at 1000 rpm. A 1 ml aliquot of cold extraction solvent [methanol/formic acid/water, 60:5:35; including 35 mg l^−1^ 5'AMP (Sigma A2252) and 250 mg l^−1^ BHT (Sigma 34750)] was added to buds (~0.3–3 mg) and then shaken for a further 2 min in the TissueLyser prior to adding an internal standard mixture containing: [^2^H_3_]dihydrozeatin (d3-DZ); [^2^H_3_]dihydrozeatin riboside (d3-DZR); [^2^H_3_]dihydrozeatin-9-glucosidase (d3-DZ9G); [^2^H_5_]*trans*-zeatin (d5-*t*Z); [^2^H_5_]*trans*-zeatin riboside (d5-*t*ZR); [^2^H_5_]*trans*-zeatin riboside-5'-monophosphate sodium salt (d5-*t*ZRP); [^2^H_5_]*trans*-zeatin-7-glucosidase (d5-*t*Z7G); [^2^H_5_]*trans*-zeatin-9-glucosidase (d5-*t*Z9G); [^2^H_5_]*trans*-zeatin-*O*-glucosidase (d5-*t*ZOG); [^2^H_5_]*trans*-zeatin-*O*-glucosidase riboside (d5-*t*ZROG); [^2^H_6_]*N*^6^ isopentenyladenine (d6-IP); [^2^H_6_]*N*^6^ isopentenyladenosine (d6-IPR); [^2^H_6_]*N*^6^ isopentenyladenosine-5'-monophosphate sodium salt (d6-IPRP); [^2^H_6_]*N*^6^ isopentenyladenine-7-glucosidase (d6-IP7G); [^2^H_6_]*N*^6^ isopentenyladenine-9-glucosidase (d6-IP9G); [^2^H_3_]dihydrophaseic acid (–)-7',7',7'-d_3_ dihydrophaseic acid (d3-DPA); [^2^H_5_]abscisic acid glucose ester (+)-4,5,8',8',8'-(d5-ABA-GE); [^2^H_3_]phaseic acid (–)-7',7',7' (d3-PA); [^2^H_4_]7'-hydroxy-abscisic acid; (±)-5,8',8',8'-d_4_-7'-hydroxy-ABA (d4-OH-ABA); [^2^H_4_]abscisic acid (–)-5,8'8'8' (d4-ABA); [^2^H_5_]indole acetic acid (d5-IAA); and [^2^H_5_]indole-3-acetylaspartic acid (d5-IAAsp) (1 ng of each compound per sample). Then, samples were left on ice in the dark for 20 min prior to centrifugation (≥10000 *g* for 10 min). The supernatants were transferred to LCMS vials, evaporated to dryness, and then dissolved in 5% acetonitrile in 10 mM ammonium formate buffer (pH 3.4). Finally, samples were filtered through 0.45 μm, 4 mm diameter syringe nylon filters.

Endogenous hormone levels were quantified by using an LC/MS-MS (triple quadrupole) instrument with an Agilent 1100 or 1200 series HPLC system (Agilent, Berks, UK) coupled to a Q-Trap 6500 mass spectrometer (AB Sciex, Framingham, MA, USA), and as described by Young *et al.* (2014) with a few modifications. The extracts (20 μl) were injected onto a Phenomenex 3 μm C_18_ Luna 100 × 2 mm with a guard column at 40 °C. The mobile phase consisted of 2% acetonitrile in 10 mM ammonium formate (A) and 95% acetonitrile in water with 0.1% formic acid (B); using a linear increase of solvent B (2% for 4 min, 16% at 20 min, and 34.5% at 25 min) at a flow rate of 200 μl min^−1^. The abundance of hormones was calculated by sample peak areas to those of corresponding deuterated standards using Analyst 1.6.2 software (AB Sciex), with manual inspection of chromatograms to ensure correct peak assignments.

Deuterated and non-deuterated ABA metabolites, (–)-DPA, (+)-ABA-GE, (–)-PA, and (±)-7'-hydroxy-ABA, were obtained from the National Research Council of Canada-Plant Biotechnology Institute; (±)-ABA was purchased from Sigma-Aldrich; and other standards were obtained from OlChemIm, Olomouc, Czech Republic.

### Statistical analysis

Statistical analysis was carried out with Genstat for Windows Version 17 Software (VSN International Ltd, Hemel Hempstead, UK). The main effects of the dormancy gene (*StCEN*) on ethylene, CO_2_, ABA, and cytokinins (CKs) were revealed by an ANOVA considering the interactions between different factors (i.e. week and line) at a probability of *P*≤0.05. Least significant difference values (LSD *P*_0.05_) have been calculated to observe mean separation. Results are significant to *P*<0.05 unless otherwise stated. Multiple comparisons analysis was carried out using the Bonferroni test in order to determine the pairwise differences between genotypes.

## Results

### Screening of *StCEN* transgenic lines for tuber sprout growth phenotypes and *StCEN* expression level

Unpublished QTL mapping data, using sprout growth data on a diploid population (06H1, Prashar *et al*., 2014), revealed a significant QTL effect on chromosome 3 (S.K. Sharma *et al*., unpublished), near a single nucleotide polymorphism (SNP) marker (c2_52371), that physically maps to ~42.4 Mb of the potato genome pseudomolecules (Sharma *et al*., 2013). A region of ~0.5 Mb either side of the SNP containing 69 genes (Supplementary Table S2) was inspected carefully for gene content, and a *CEN* gene (*StCEN*, which maps to ~42.5 Mb on chromosome 3) was identified as a candidate gene for being the causative factor underlying the QTL. The *StCEN* alleles from the 06H1population parents [HB171(13) and 99FT1b5)] were cloned following PCR amplification from genomic DNA and sequenced. The 99FT1b5 parent is heterozygous for *StCEN* and contained two different alleles to HB171(13) that is homozygous for *StCEN*. The deduced amino acid sequences of all three alleles, however, were identical (Supplementary Fig. S2). Using the constitutive 35S CaMV promoter, gene constructs were developed with the aim of overexpressing this gene or reducing its expression level by RNAi, in transgenic lines of the potato variety Désirée.

**Fig. 1.**
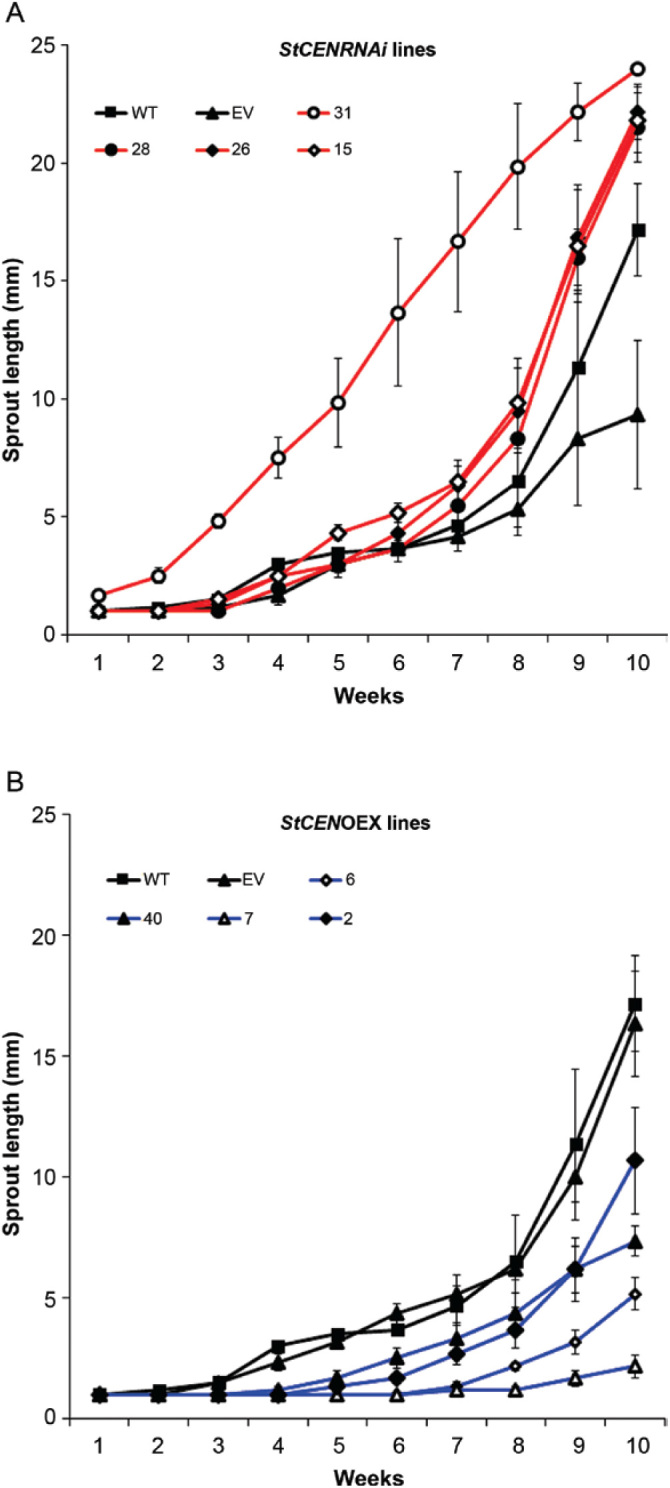
Comparison of sprout length (rounded to the nearest millimetre) of wild-type (WT) Désirée with transgenic plants either (A) down-regulating (RNAi) or (B) overexpressing (OEX) the *StCEN* gene. Empty vector (EV) transformed control is also included for comparison. Error bars represent the SE of six biological replicates.

Tubers from the transgenic lines (four OEX lines and four RNAi lines) were assessed for tuber sprout growth compared with controls [wild-type Désirée (WT) and an empty vector transformant]. Whereas all OEX lines exhibited a lower rate of sprout growth than controls, RNAi lines showed an increase in sprout growth rate (Fig. 1).

**Fig. 2.**
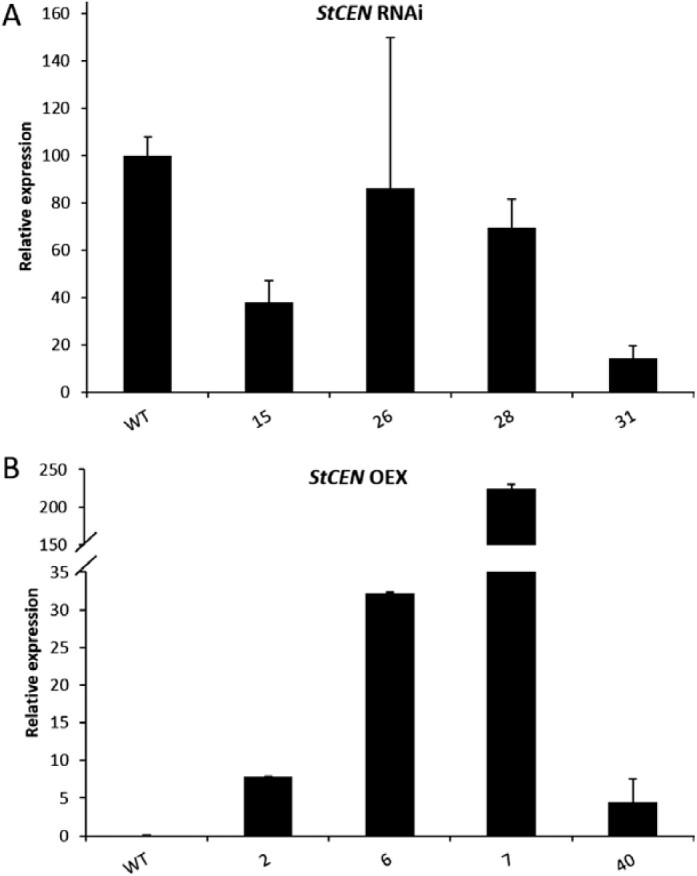
Comparison of gene expression levels in sprouts of wild-type (WT) Désirée with transgenic plants either (A) down-regulating (RNAi) or (B) overexpressing (OEX) the *StCEN* gene as determined by quantitative PCR. Error bars represent the SE of three biological replicates.

**Fig. 3.**
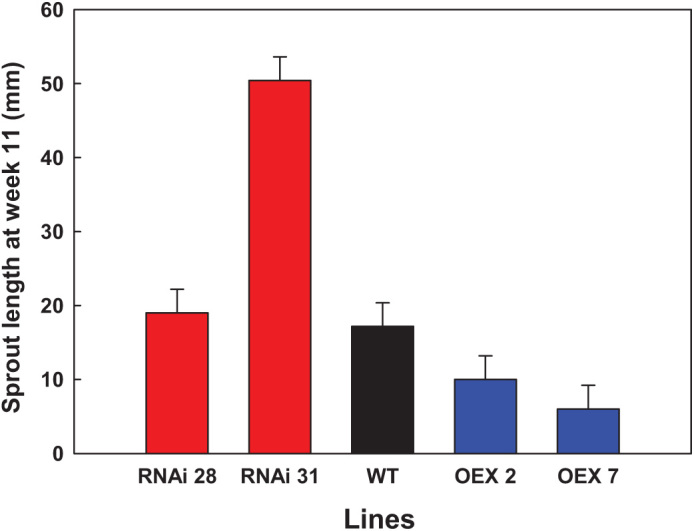
Sprout length (mm) after 11 weeks of cold storage (10 °C) for wild-type (WT - Désirée) tubers and *StCEN* transgenic potatoes, where RNAi28 and RNAi31 are silenced lines and OEX2 and OEX7 are overexpressed lines. For each genotype, four tubers from three individual plants were studied. Histogram bars correspond to the mean of three replicates per line, where four tubers per replicate were assessed. LSD bars (*P*<0.05) are shown.

*StCEN* expression level was measured in buds/subtending parenchyma by qRT-PCR (Fig. 2). For overexpressing lines, the *StCEN* expression level in bud tissues was higher, with the maximum expression level measured in line 7 at ~225-fold higher levels than in controls (Fig. 2B). This line also exhibited the slowest rate of sprout growth (Fig. 3). To assess the *StCEN* expression level in RNAi lines, a sensitive assay using SYBR^®^ green was developed. The lowest bud *StCEN* expression levels were measured in lines 27 and 31 at ~15% of the level in the WT (Fig. 2A). Line 31 exhibited the highest tuber sprout growth rate (Fig. 3).

### Dormancy phenotyping of *StCEN* transgenic lines

Based on the phenotypic screening of the transgenic lines and *StCEN* expression level, two *StCEN* OEX lines (line 7 highly overexpressing, line 2, weakly overexpressing) and two *StCEN* RNAi lines (line 31, strongly reduced, and line 28, weakly reduced *StCEN* expression) as well as WT controls were selected for detailed analysis in potato plants grown from a subsequent tuber generation. Whilst similar trends were observed for the transgenic lines tested in the two tuber generations, there were some differences in the time taken from harvest to observable tuber sprouting. The dormancy phenotyping (Table 1) confirmed that OEX, RNAi, and WT lines were not differentiated according to time to dormancy break (EM). For example, RNAi line 28 and OEX line 2 had partially broken dormancy at the same time (after 1 week of storage), with all replicates showing eye movement in the third week, whilst WT tubers remained dormant the longest (until the fifth week).

**Table 1.**
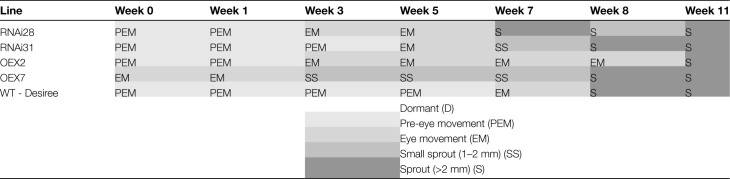
Dormancy break and sprout growth assessment for wild-type (WT - Désirée) and *StCEN* transgenic potatoes, where RNAi28 and RNAi31 are silenced lines and OEX2 and OEX7 are overexpressed lines

A greyscale was used to represent each phenotyping category. D, dormant; PEM, pre-eye movement; EM, eye-movement (=dormancy break); SS, small sprout (1–2 mm); S, sprout (>2 mm). For each genotype and sampling point, five tubers from three individual plants were studied (except from week 11 where four tubers per genotype and replicate were considered). The assignment of a specific phenotyping category is based on three replicates.

As well as there being no consistent difference in dormancy break, based on the *StCEN* expression level, the transgenic lines and controls did not show a clear pattern in the time taken for initial sprout growth. Thus, OEX line 7 initiated sprouts in week 3, followed by RNAi line 28, 31, and the WT in week 7. However, OEX line 2 had not initiated sprouts by week 8. In contrast to the effects on dormancy release and sprouting onset, an ANOVA test demonstrated a clear effect of *StCEN* expression level on sprout vigour after 11 weeks of storage at 20 °C. The overexpressing lines had significantly shorter sprouts (*P*<0.05) than both WT and RNAi lines. Conversely, RNAi line 31 (strongly reduced *StCEN* expression) tubers had the longest sprout length (*P*<0.05) while RNAi line 28 (weakly reduced *StCEN* expression) and WT had similar values (Fig. 3; Supplementary Fig. S3).

### Respiration rate and ethylene production of *StCEN* transgenic lines

**Fig. 4.**
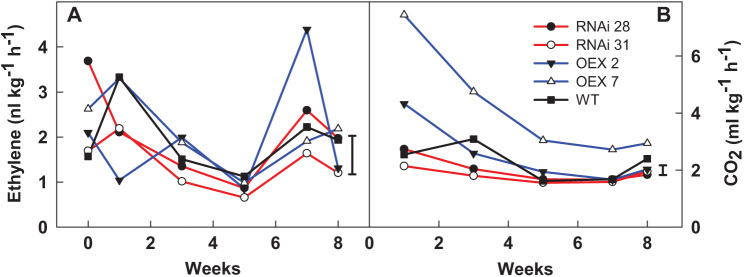
(A) Ethylene (nl kg^−1^ h^−1^) and (B) CO_2_ (ml kg^−1^ h^−1^) production of wild-type (WT - Désirée) tubers and *StCEN* transgenic potatoes throughout post-harvest storage (8 weeks) at 20 °C. For each genotype, four tubers from three individual plants were studied. Data points correspond to the mean of three replicate measurements; five tubers per replicate were considered. LSD bars (*P*<0.05) for the interaction weeks×line are shown.

The overexpression or silencing of *StCEN* did not significantly affect endogenous ethylene production of potato tubers throughout storage at 20 °C. However, a significant (*P*<0.05) decrease in ethylene production was observed from the first to the fifth week of storage in all the lines, preceding sprout onset with rates at a minimum at week 5 (0.7–1.1 nl h^−1^ kg^−1^) (Fig. 4a).

Overexpressing lines experienced a 3-fold decrease, whereas silenced lines had almost a 2-fold decrease (Fig. 4a).

The respiration rate of one of the overexpressed lines (OEX line 7) was significantly higher than that of the control (WT) throughout storage, while the silenced lines showed the lowest CO_2_ production (Fig. 4b) At week 8, a small increase accompanied sprout onset, regardless of the line, yet was only significantly higher for the WT.

### Significant effects on ABA and CK metabolites in *StCEN* transgenic lines

**Fig. 5.**
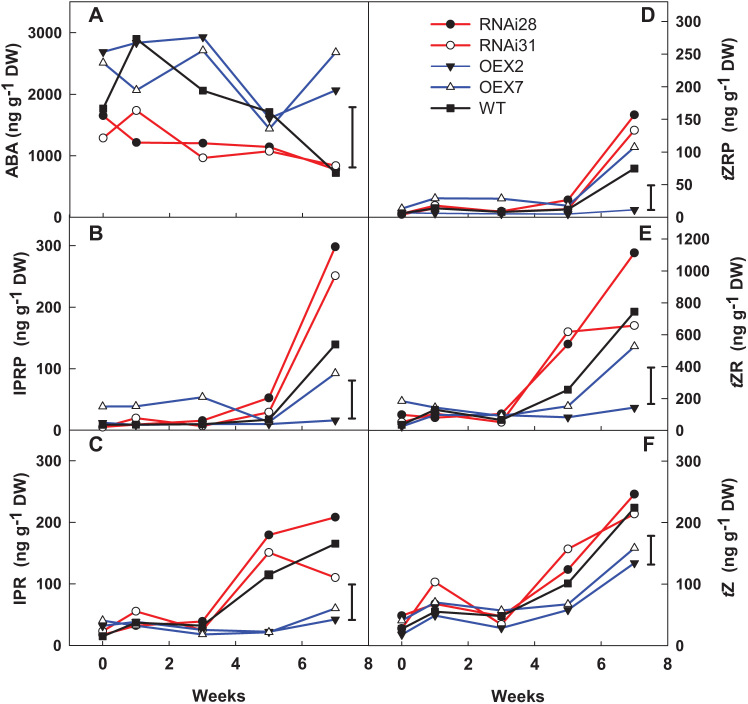
(A) ABA and cytokinins (B) IPR-5'-monophosphate (IPRP), (C) isopentenyl-adenosine (IPR), (D) *t*ZR-5'-monophosphate (*t*ZRP), (E) *t*Z-riboside (*t*ZR), and (F) *trans*-zeatin (*t*Z)] concentrations as ng g^−1^ per DW in *StCEN* potato apical buds. Results are from a post-harvest storage experiment at 20 °C for 7 weeks, where RNAi28 and RNAi31 are silenced lines and OEX2 and OEX7 are overexpressed lines. For each genotype, four tubers from three individual plants were studied. Data points correspond to the mean of three replicate measurements; five tubers per replicate were considered. LSD bars (*P*<0.05) are shown.

The meristematic tissue of overexpressing lines exhibited a significantly higher (*P*<0.05) ABA concentration (~1500–3000 ng g^−1^ DW) in buds throughout the 7 weeks of analysis compared with silenced lines (~750–1700 ng g^−1^ DW). Furthermore, the WT showed similar concentrations in buds to those of overexpressing lines at the beginning of storage, whereas the concentration in week 7 was at the same level as that of silenced lines (Fig. 5).

Overall silenced lines tended to have higher CK concentrations in buds (Fig. 5). Levels of isopentenyladenosine (IPR) and its precursor [isopentenyladenosine-5'-monophosphate (IPRP)] increased (*P*<0.05) at sprout onset (week 7) in control lines but not in samples from overexpressing lines. This increase was significantly higher in silenced lines. Therefore, IPRP appears to correlate inversely with *StCEN* expression. Abundance was similar for both CKs and increased from 5–20 ng g^−1^ DW to 100–300 ng g^−1^ DW in the case of silenced lines and from 10–40 ng g^−1^ DW to 20–40 ng g^−1^ DW for overexpressing lines.

A similar tendency was found for the bioactive CK *t*Z and its precursors (*t*ZR and *t*ZRP) in buds, with a period of stability followed by an increased level after sprouting onset. Silenced lines significantly increased these CKs from week 5 (*t*ZRP) or week 3 (*t*Z and *t*ZR), whereas overexpressing lines showed a significantly lower rise.

## Discussion

### Identification of *StCEN* as a candidate gene for the control of tuber sprouting

*StCEN* was identified as a candidate gene for the control of tuber sprouting as the gene underlies a large effect QTL for sprout growth identified in a previous study (S.K. Sharma *et al*., unpublished). In the previous study, a biparental population of ~180 diploid genotypes was analysed for tuber sprout growth in storage. As tuber bud dormancy release is a prerequisite for sprout growth, the phenotypic analysis was not designed to discriminate between dormancy release and sprout growth. The QTL identified, therefore, could impact on dormancy release or sprout growth, or both processes. Although 69 genes were present within the QTL limit, *StCEN* was an attractive candidate for being the causative gene, based on the known effects of *TFL1/CEN* homologues on bud maturation and outgrowth in several species (Mohamed *et al.*, 2010; Varkonyi-Gasic *et al*., 2013). *StCEN* is a member of the extended FT family of 13 genes identified in the potato genome (Abelenda *et al.*, 2014). Members of this gene family include *SP6A* and *SP5G*, identified as key regulators of tuber initiation. *StSP6A* and *FT* encode small globular proteins belonging to the phosphatidylethanolamine-binding protein (PEBP) family. In Arabidopsis and rice, FT has been shown to interact in the shoot apex with the basic leucine zipper (bZIP) transcriptional factor FLOWERING LOCUS D (FD) forming a complex, together with a 14-3-3 protein, termed the florigen activation complex (FAC). The interaction of these proteins leads to the activation of several MADS-box floral identity genes such as *APETALA1* (*AP1*) and the initiation of floral induction (Abe *et al.*, 2005; Wigge *et al.*, 2005; Taoka *et al.*, 2011). FD also interacts with the flowering repressor TERMINAL FLOWER-1 (TFL1), homologous to CENTRORADIALIS (CEN), forming a transcriptional inhibitory complex that represses the same floral identity genes that are activated by FT (Hanano and Goto, 2011). This indicates that FD is required for TFL1 activity and that FT and TFL1 function antagonistically (Hanano and Goto, 2011). Recently a complex termed the tuberigen activation complex (TAC) comprised of StSP6A, St14-3-3s, and StFLOWERING LOCUS D-LIKE 1 (StFDL1) has been identified (Teo *et al*., 2017). The TAC is thought to have a regulatory role in tuber initiation acting in an analogous manner to the FAC for flowering. A further member of this gene family, *SP3D*, is thought to encode an important signal controlling flowering (Navarro *et al.*, 2011). The functions of other FT family members remain to be elucidated but, based on studies in other plant species, these are likely to be important in the regulation of wide-ranging developmental processes.

### *StCEN* transgenic lines show tuber sprout growth effects due to altered sprout vigour rather than effects on tuber bud dormancy release

A set of transgenic lines was developed that constitutively overexpressed *StCEN* or exhibited decreased levels of *StCEN* expression. Preliminary screening experiments demonstrated a clear effect of manipulation of *StCEN* expression, with decreased levels associated with an increased rate of sprout growth and overexpressing lines showing a lower rate of sprout growth than controls (Figs 1, 2). Generally, in transgenic lines where the *StCEN* expression level was most changed, there was the strongest phenotype (for either overexpressing or reduced expression lines), providing evidence linking phenotype to the *StCEN* expression level. Detailed analysis of dormancy release and the onset of tuber sprouting indicated that there was no clear trend in the timing of dormancy release between the transgenic lines, despite the major difference in subsequent sprout growth rate. Thus, we conclude that the *StCEN* expression level is a major determinant in the control of tuber sprout growth rate, yet has little effect on tuber bud dormancy release. From a commercial perspective, reduction in sprouting during storage is an important aim, whether by extending the dormancy period or by reducing sprout growth post-dormancy release. As the *StCEN* candidate was identified in a genetic screen, the implication is that alleles of *StCEN* exist that will impact on tuber sprout growth. Additionally, as all three alleles identified in the diploid parents of the cross encode identical proteins, allelic effects are most likely to be due to differences in expression levels. Further work will be required to characterize these alleles and determine whether markers can be developed that would find utility in a breeding programme for developing potato genotypes with improved storage characteristics.

### *StCEN* expression is associated with ABA and CK levels in tuber buds

In order to learn more about how *StCEN* elicits its effects on tuber sprout growth, the levels of key plant growth regulators were compared in tuber buds from the transgenic lines and control during dormancy release and the onset of sprouting.

Suttle, 1998*a* showed that endogenous ethylene is necessary for the initiation of tuber dormancy. Endogenous ethylene production from *StCEN* tubers was in general not significantly different from that in controls, perhaps indicating the lack of effect of *StCEN* expression level on dormancy *per se*.

The decrease in ABA levels found in apical buds throughout post-harvest is in accordance with previous work and supports the hypothesis that ABA is required for both the initiation and maintenance of dormancy (Suttle and Hultstrand, 1994; Sonnewald, 2014; Foukaraki *et al*., 2016*a*). However, the existence of an ABA threshold below which dormancy is broken or sprouting is initiated remains unsupported by the current study. As in the study of Sonnewald (2001), no correlations were found between ABA concentration levels and the specific timing of dormancy break or sprout onset in buds.

Significantly higher ABA levels were observed in the buds from overexpressing lines compared with silenced lines. Moreover, the declining trend of ABA was less clear for overexpressing lines, maintaining a high concentration difference with silenced lines even after sprouting. This suggests that *StCEN* overexpression may produce a rise in ABA production and, as a consequence, and in combination with higher levels of CKs (see below), a reduction in sprout growth/vigour.

The concentration of CKs was generally higher in *StCEN* silenced lines during the whole period of storage but this difference was emphasized after sprouting occurred. WT levels of CKs remained intermediate between both OEX and reduced expression transgenic lines, indicating a negative link between *StCEN* expression level and CK synthesis. Thus, the hypothesized role of *StCEN* in reducing sprout growth is supported by the reduced CK content of the overexpressed lines.

The concentration of CKs in apical buds increases markedly with the onset of sprouting, as previously shown by other studies (Hartmann *et al.*, 2011; Suttle, 1998*b*, 2008. Using a transgenic approach in potato lines with reduced CK content due to expression of an Arabidopsis *cytokinin oxidase/dehydrogenase1* gene, Hartmann *et al.* (2011) demonstrated the requirement of CK for dormancy release. Exogenous application of CK resulted in dormancy release, but subsequent sprout growth required the additional application of gibberellins. Interestingly, the link between CK level and expression of FT family members has been reported previously in Arabidopsis (D’Aloia *et al.*, 2011).

Within the scope of the current study, not all plant growth regulators were measured as the focus was on ABA and CKs, most closely associated with dormancy break. In view of the effects on sprout growth, the effects of *CEN* expression manipulation on gibberellin levels would be of particular interest. Auxin and strigolactones are also implicated in dormancy release (reviewed in Alamar *et al.*, 2017) and so further work on the *CEN* transgenic lines may clarify their potential roles in potato tuber dormancy release and sprouting.

In conclusion, the *StCEN* expression level had no consistent effect on time to potato tuber dormancy break but did influence sprout vigour, where *StCEN* expression was inversely correlated with the rate of sprout growth. These findings are intriguing as *FT* gene family members are developmental regulators with critical roles in tuberization. Here we show that a specific *FT* family member is also involved in tuber sprouting, another important aspect of the tuber life cycle.

## Supplementary Data

Supplementary data are available at *JXB* online.

Table S1. Primers used for cloning and qRT-PCR analysis

Table S2. List of genes within 0.5 Mb of the chromosome 3 SNP marker c2_52371.

Fig. S1. Dormancy phenotyping. Each of the images corresponds to one of the states within the bud growth classification developed herein.

Fig. S2. Sequence analysis of *StCEN* alleles.

Fig. S3. Sprouting phenotypes of *StCEN* transgenic lines and controls after 11 weeks storage.

Supplementary Table S1

Supplementary Table S2

Supplementary Figures S1-S3
