# Supplementary material for: A member of the TERMINAL FLOWER 1/CENTRORADIALIS gene family controls sprout growth in potato tubers
Source: J Exp Bot. 2018 Nov 3;70(3):835–43. doi: 10.1093/jxb/ery387 (PMC6363080; doi:10.1093/jxb/ery387)
Supplement: Supplementary Table S1 [file ery387_suppl_supplementary_table_s1.pdf]

Supplementary Table S1. Primer sequences used in this study.

Gene	PGSC ID	Purpose	Forward	Reverse
StEF1a	PGSC0003DMT400059830	QPCR	CTTGACGCTCTTGACCAGATT	GAAGACGGAGGGGTTTGTCT
StCEN	PGSC0003DMT400037143	QPCR of OEX	TGGGAGCAAACAAGTTTCTAATG	TTCGACACGAGGTTGAGTTG
StCEN	PGSC0003DMT400037143	QPCR of RNAi	AATGCCCAGAGAGAAACTGC	ATTTTGTGTGTGTGTGTGTCAAAT
StCEN	PGSC0003DMT400037143	Cloning of OEX	AAGTCGACAAAAATGTCTTCTAGAGGTACTTG	AAGTCGACAAATTCATCTTCTTCTAGCTGCAG
StCEN	PGSC0003DMT400037143	Cloning of RNAi	ACCAGGTCTCAGGAGCCACTTGCAGTAGGGAGAGT	ACCAGGTCTCATCGTCCAATCCATTTTCCGCCGAA
