# Supplementary material for: A member of the TERMINAL FLOWER 1/CENTRORADIALIS gene family controls sprout growth in potato tubers
Source: J Exp Bot. 2018 Nov 3;70(3):835–43. doi: 10.1093/jxb/ery387 (PMC6363080; doi:10.1093/jxb/ery387)
Supplement: Supplementary Figures S1-S3 [file ery387_suppl_supplementary_figures_s1-s3.pdf]

Supplementary Figures

A member of the *TERMINAL FLOWER 1/CENTRORADIALIS* gene family controls sprout growth in potato tubers.

Wayne L. Morris[1], M. Carmen Alamar[2], Rosa M. Lopez-Cobollo[3], Javier Cañete Castillo[2], Mark Bennett[2], Jeroen Van der Kaay[1], Jennifer Stevens[1], Sanjeev Kumar Sharma[1], Karen McLean[1], Andrew J. Thompson[2], Leon A. Terry[2], Colin G.N. Turnbull[3], Glenn J. Bryan[1], Mark A. Taylor[1]

Supplementary Figure Legends

Figure S1. Dormancy phenotyping. Each of the images corresponds to one of the states within the bud growth classification developed herein. Where (A) is dormant [D]; (B) pre- eye movement [PE]; (C1 and C2) eye movement [EM]; (D) small sprout [SS] (1-2 mm); (E) sprout [S] (>2 mm). Scale bar: 1mm. Dashed circles indicate the eye position; black lines, the primordial leaves; and green lines, the buds.

Figure S2. Allelic variation of *Centroradialis* (*StCEN*) gene. A. Clustal Omega alignment of the genomic DNA sequences of *CEN* alleles 99.FT.1#1, 99.FT.1#2, and HB.171.1#1 isolated from parents of the 06H1 population (HB171(13) and 99FT1b5). Exons and Introns are indicated by red and black text, respectively. B. Clustal Omega alignment of the translated protein sequence of the three CEN alleles. Clustal Omega program is available at https://www.ebi.ac.uk/Tools/msa/clustalo/.

Figure S3. Image showing the sprout length for *StCEN* overexpressed (OEX2, OEX7) and underexpressed (RNAi28, RNAi31) lines, and wild type (WT) tubers after 11 weeks at 20 °C, compared to week 0.

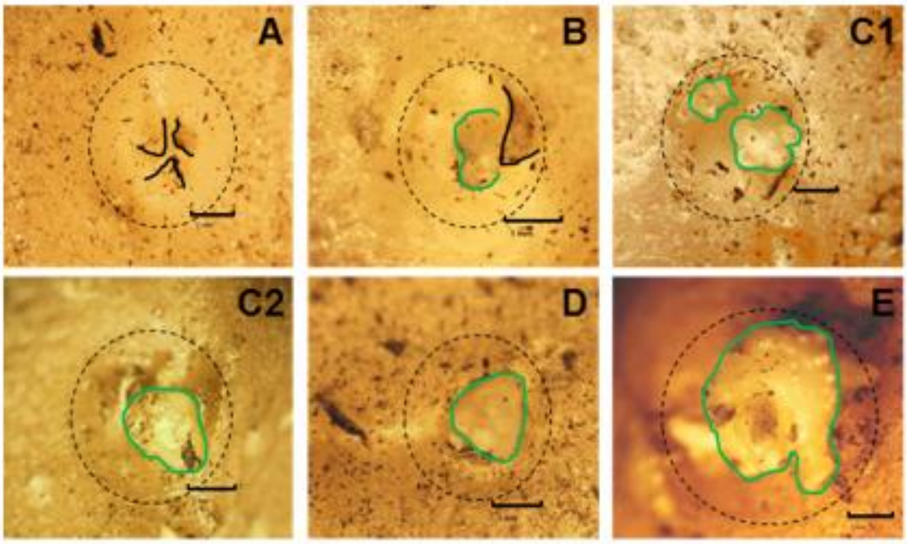

Figure S1. Dormancy phenotyping. Each of the images corresponds to one of the states within the bud growth classification developed herein. Where (A) is dormant [D]; (B) pre- eye movement [PE]; (C1 and C2) eye movement [EM]; (D) small sprout [SS] (1-2 mm); (E) sprout [S] (>2 mm). Scale bar: 1mm. Dashed circles indicate the eye position; black lines, the primordial leaves; and green lines the buds.

A

```
99.FT.1_CEN#1      TCATCTTCTTCTCGCTGCAGTTTCTCTCTGGGCATTGAAATAAACAGCAGCAACAGGGGA    60
HB.171.1_CEN#1     TCATCTTCTTCTAGCTGCAGTTTCTCTCTGGGCATTGAAATAAACAGCAGCAACAGGGGA    60
99.FT.1_CEN#2      TCATCTTCTTCTAGCTGCAGTTTCTCTCTGGGCATTGAAATAAACAGCAGCAACAGGGGA    60
                   ************ ***********************************************

99.FT.1_CEN#1      TCCCAATCCATTTTCCGCCGAAAAACTCCTTGTGTTGAATTGATCCCTTGTTGATGGTGC    120
HB.171.1_CEN#1     TCCCAATCCATTTTCCGCCGAAAAACTCCTTGTGTTGAATTGATCCCTTGTTGATGGTGC    120
99.FT.1_CEN#2      TCCCAATCCATTTTCCGCCGAAAAACTCCTTGTGTTGAATTGATCCCTTGTTGATGGTGC    120
                   ************************************************************

99.FT.1_CEN#1      TTTCACTGTTTGCCTTCCTCTTTGTTTATACAACAAAAATACATAACGATGTATCCCAAT    180
HB.171.1_CEN#1     TTTCACTGTTTGCCTTCCTCTTTGTTTATACAACAAAAATACATAACGATGTATCCCAAT    180
99.FT.1_CEN#2      TTTCACTGTTTGCCTTCCTCTTTGTTTATACAACAAAAATACATAACGATGTATCCCAAT    180
                   ************************************************************

99.FT.1_CEN#1      TACCGGCTTTGGTGTCTCGTAGCACACTATCTCCCTTCCTGTAAATCACAAATTTAAAAT    240
HB.171.1_CEN#1     TACCGGCTTTGGTGTCTCGTAGCACACTATCTCCCTTCCTGTAAATCACAAATTTAAAAT    240
99.FT.1_CEN#2      TACCGGCTTTGGTGTCTCGTAGCACACTATCTCCCTTCCTGTAAATCACAAATTTAAAAT    240
                   ************************************************************

99.FT.1_CEN#1      TAAATTATACACGATTCACCAGTTGCGTGACCATCTGAAAAGTCAACTCGAGAATCAAAC    300
HB.171.1_CEN#1     TAAATTATACACGATTCACCAGTTGCGTGACCATCTGAAAAGTCAACTCGAGAATCAAAC    300
99.FT.1_CEN#2      TAAATTATACACGATTCACCAGTTGCGTGAACATCTGAAAAGTCAACTCGAGAATCAAAC    300
                   ****************************** *****************************

99.FT.1_CEN#1      TTACCAAAAGAAATGTCAGTTGTACCGGGAATGTCTGTCACAATCCTGATATTATAAGCC    360
HB.171.1_CEN#1     TTACCAAAAGAAATGTCAGTTGTACCGGGAATGTCTGTCACAATCCTGATATTATAAGCC    360
99.FT.1_CEN#2      TTACCAAATGAGATGTCAGTTGTACCAGGAATGTCTGTCACAATCCTGATATTATGAACC    360
                   ******** ** ************** **************************** * **

99.FT.1_CEN#1      CATAATTTAGTCAACATAAGAAACACTTGTACTAATATTGTAAAATATTACATCAATTAA    420
HB.171.1_CEN#1     CATAACTTAGTCAACATAATAAACACTTGTACTAATATTGT---ATATTACATCAATTAA    417
99.FT.1_CEN#2      CATAATTTAGTCAACATAAGAAACACTTGTACTAATATTGT---ATATTACATCAATTAA    417
                   ***** ************* *********************   ****************

99.FT.1_CEN#1      TTTGAATTATCATCAGATTTTAGAATAAGAAATATTACCAATGGAGATGTTCCCTCAAGT    480
HB.171.1_CEN#1     TTTGAATTATCATCAGATTTTAGAATAAGAAATATTACCAATGGAGATGTTCCCTCAAGT    477
99.FT.1_CEN#2      TTTGAATTATCATCAGATTTTAGAATAAGAAATATTACCAATGGAGATGTTCCCTCAAGT    477
                   ************************************************************

99.FT.1_CEN#1      ATGGATCACTAGGACTTGGAGCATCAGGGTCCGTCATGATCTTCCCAAAGAAAAAAAGAC    540
HB.171.1_CEN#1     ATGGATCACTAGGACTTGGAGCATCAGGGTCCGTCATGATCTTCCCAAAGAAAAAAAGAC    537
99.FT.1_CEN#2      ATGGATCACTAGGACTTGGAGCATCAGGGTCCGTCATGATCTTCCCAAAGAAAAAAAGAC    537
                   ************************************************************

99.FT.1_CEN#1      AACAATCACAAACGGAATACAAAACAACAAATACAATGTACAAACAT-----AAACTCTG    595
HB.171.1_CEN#1     AACAATCAGAAACGGAATACAAAACAACAAATACAATGTACAAACATAACATAACCTCTG    597
99.FT.1_CEN#2      AACAATCAGAAACGGAATACAAAACAACAAATACAATGTACAAACATAACATAACCTCTG    597
                   ******** **************************************     ** *****

99.FT.1_CEN#1      TTTCATTTTATATAAACACAAACTTCGTGGTCAGTCAAACACA------CATATGTATGT    649
HB.171.1_CEN#1     TTTCATTTTATATAAACACAAACTTCGTGGTCAGTCAAACACACACGTATGTATGTATGT    657
99.FT.1_CEN#2      TTTCATTTTATATAAACACAAACTTCGTGGCCAGTCAAACA--CACGTATGTATGTATGT    655
                   ****************************** **********          *********

99.FT.1_CEN#1      ATGAGACGACTTACGAGTGTATAAGCAGATCTCATGTCTTCACCACCAATTTCGACACGA    709
HB.171.1_CEN#1     ATGAGACGACTTACGAGTGTATAAGCAGATCTCATGTCTTCACCACCAATTTCGACACGA    717
99.FT.1_CEN#2      ATGAGACGACTTACGAGTGTATAAGCAGATCTCATGTCTTCACCACCAATTTCGACACGA    715
                   ************************************************************

99.FT.1_CEN#1      GGTTGAGTTGCAACAACAGCAGGCATGATCTCATGGCCATTAGAAACTTGTTTGCTCCCA    769
HB.171.1_CEN#1     GGTTGAGTTGCAACAACAGCAGGCATGATCTCATGGCCATTAGAAACTTGTTTGCTCCCA    777
99.FT.1_CEN#2      GGTTGAGTTGCAACAACAGCAGGCATGATCTCATGGCCATTAGAAACTTGTTTGCTCCCA    775
                   ************************************************************

99.FT.1_CEN#1      TTGTATATAACTTTCATTTTCACACTTGGACTAAAACTGTCTACTACTTCACCTATTACT    829
HB.171.1_CEN#1     TTGTATATAACTTTCATTTTCACACTTGGACTAAAACTGTCTACTACTTCACCTATTACT    837
99.FT.1_CEN#2      TTGTATATAACTTTCATTTTCACACTTGGACTAAAACTGTCTACTACTTCACCTATTACT    835
                   ************************************************************

99.FT.1_CEN#1      CTCCCTACTGCAAGTGGTTCACAAGTACCTCTAGAAGACAT    870
HB.171.1_CEN#1     CTCCCTACTGCAAGTGGTTCACAAGTACCTCTAGAAGACAT    878
99.FT.1_CEN#2      CTCCCTACTGCTAGTGGTTCACAAGTACCTCTAGAAGACAT    876
                   *********** *****************************
```

B

```
CLUSTAL O(1.2.4) multiple sequence alignment


99.FT.1_CEN#1       MSSRGTCEPLAVGRVIGEVVDSFSPSVKMKVIYNGSKQVSNGHEIMPAVVATQPRVEIGG      60
99.FT.1_CEN#2       MSSRGTCEPLAVGRVIGEVVDSFSPSVKMKVIYNGSKQVSNGHEIMPAVVATQPRVEIGG      60
HB.171.1_CEN#1      MSSRGTCEPLAVGRVIGEVVDSFSPSVKMKVIYNGSKQVSNGHEIMPAVVATQPRVEIGG      60
                    ************************************************************

99.FT.1_CEN#1       EDMRSAYTLIMTDPDAPSPSDPYLREHLHWIVTDIPGTTDISFGREIVCYETPKPVIGIH      120
99.FT.1_CEN#2       EDMRSAYTLIMTDPDAPSPSDPYLREHLHWIVTDIPGTTDISFGREIVCYETPKPVIGIH      120
HB.171.1_CEN#1      EDMRSAYTLIMTDPDAPSPSDPYLREHLHWIVTDIPGTTDISFGREIVCYETPKPVIGIH      120
                    ************************************************************

99.FT.1_CEN#1       RYVFLLYKQRGRQTVKAPSTRDQFNTRSFSAENGLGSPVAAVYFNAQRETAARRR* 175
99.FT.1_CEN#2       RYVFLLYKQRGRQTVKAPSTRDQFNTRSFSAENGLGSPVAAVYFNAQRETAARRR* 175
HB.171.1_CEN#1      RYVFLLYKQRGRQTVKAPSTRDQFNTRSFSAENGLGSPVAAVYFNAQRETAARRR* 175
                    *******************************************************
```

Figure S2. Allelic variation of *Centroradialis* (*StCEN*) gene. A. Clustal Omega alignment of the genomic DNA sequences of *CEN* alleles 99.FT.1#1, 99.FT.1#2, and HB.171.1#1 isolated from parents of the 06H1 population (HB171(13) and 99FT1b5). Exons and Introns are indicated by red and black text, respectively. B. Clustal Omega alignment of the translated protein sequence of the three CEN alleles. Clustal Omega program is available at https://www.ebi.ac.uk/Tools/msa/clustalo/.

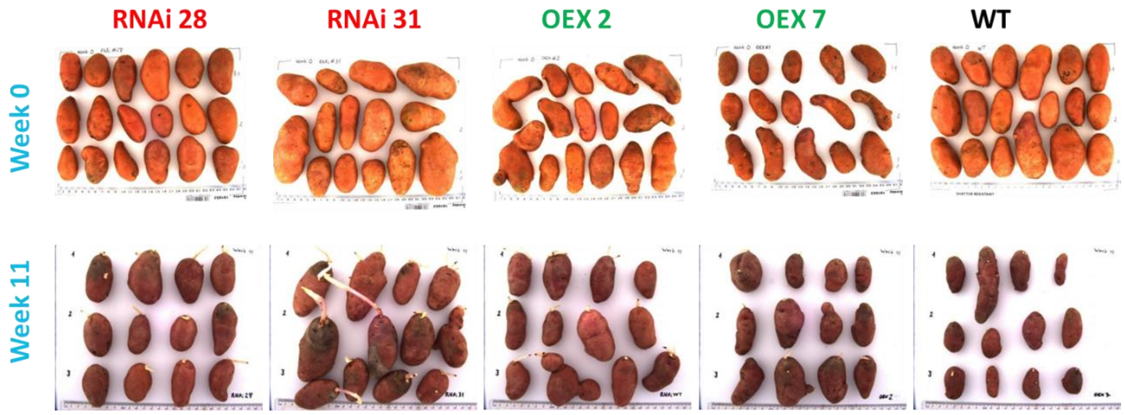

Figure S3. Image showing the sprout length for *StCEN* overexpressed (OEX2, OEX7) and underexpressed (RNAi28, RNAi31) lines, and wild type (WT) tubers after 11 weeks at 20 °C, compared to week 0.
